# Post liver transplant metabolic syndrome: Frequency, predictors and outcome

**DOI:** 10.12669/pjms.41.2.10774

**Published:** 2025-02

**Authors:** Zoha Rahim, Kashif Malik, Shahid Sarwar, Adnan Salim

**Affiliations:** 1 Zoha Rahim, MBBS, MD (Gastroenterology) Assistant Professor Gastroenterology, Allama Iqbal Medical College Lahore, Lahore, Pakistan; 2 Kashif Malik, MBBS, FCPS (Gastroenterology) Professor of Gastroenterology & Hepatology, Allama Iqbal Medical College Lahore, Pakistan, Shaikh Zayed Post Graduate Medical Institute, Lahore, Pakistan; 3 Shahid Sarwar, MBBS, FCPS (Medicine), FCPS (Gastroenterology), MCPS-HPE, FRCP (Edin) Professor of Medicine & Gastroenterology, Allama Iqbal Medical College Lahore, Lahore, Pakistan; 4 Adnan Salim, MBBS, FCPS (Gastroenterology) Assistant Professor of Gastroenterology, Shaikh Zayed Post Graduate Medical Institute, Lahore, Pakistan

**Keywords:** Cardiovascular events, Liver transplantation, Post-transplant metabolic syndrome, Predictors

## Abstract

**Objective:**

To determine frequency of post-transplant metabolic syndrome (PTMS) after liver transplantation (LT), its pre-transplant predictors and its association with cardiovascular events.

**Methods:**

In this observational, analytical cross-sectional study done at Gastroenterology Department, Shaikh Zayed Post Graduate Medical Institute Lahore from January 2021 to March 2023, pre-transplant data of patients having LT for > 1 year including etiology of liver disease, presence of metabolic syndrome (MS), diabetes mellitus (DM), hypertension (HTN) and obesity were noted. Post-transplant evaluation was done to document DM, HTN, Dyslipidemia, obesity, PTMS and cardiovascular events after LT. Student’s t test and chi square were used for correlation and linear regression for multivariate analysis.

**Results:**

Total of 111 post LT patients with mean age 45.2 (±10.45) and male to female ratio 6.4/1 (96/15) were included. Before LT, 15(13.5%) patients had DM, 11 (9.9%) had HTN, 48(43.2%) were obese and MS was present in 13 (11.7%) patients. Median duration since LT was three years. Post LT, PTMS developed in 60(54.1%) patients, 64(57.7%) had DM, 27(24.3%) had HTN, dyslipidemia was noted in 60(54.1%) patients and 69(62.2%) were obese. Presence of DM (OR 15.21; p< 0.001), HTN (OR 10.00; p=0.01) and MS (OR 12.50; p=0.003) before transplant was significantly associated with development of PTMS. No significant difference in development of cardiovascular events was noted in patients with and without PTMS.

**Conclusion:**

Post transplant metabolic syndrome develops in majority of LT patients, risk is higher in those with diabetes mellitus, hypertension and metabolic syndrome before liver transplantation.

## INTRODUCTION

Metabolic syndrome (MS) is a complex obesity related disorder characterized by co-existence of obesity, diabetes mellitus, hypertension and dyslipidemia.^1^ With changing dietary habits and increasingly sedentary life style, prevalence of metabolic syndrome is on rise across all over the world. It is reported to have implicated 34% of western population with more than 40% prevalence in those above 60 years of age.^2^ Patients with diabetes mellitus have prevalence of MS as high as 85.5%.^3^ Major long term risk of MS is progressive atherosclerosis resulting in myocardial infarction, transient ischemic attack (TIA) or cerebrovascular accident (CVA).^4^

Non-alcoholic fatty liver disease (NAFLD) is a common hepatic manifestation of MS which can progress through persistent inflammation to cirrhosis and end stage liver disease.^5^ Just like MS, we are witnessing an exponential rise in prevalence of NAFLD in last few decades turning it in to a global epidemic. Prevalence of NAFLD has increased from 25.5% before 2005 to 37.8% after 2016.^6^ First liver transplant for NAFLD was done in 1996, however now NAFLD is the leading cause of liver transplantation in women and population above 65 years of age and is likely to be leading indication for all liver transplantations by year 2030.^7^

Patients who are transplanted for NAFLD related cirrhosis have higher risk of post-transplant metabolic syndrome (PTMS). PTMS is seen in 25% of transplanted patients with prevalence as high as 60% in those transplanted for NAFLD.^8^ PTMS can be either recurrent, where it was present before transplant as well, or de. Novo, new onset after liver transplantation.^9^ Development of PTMS can mitigate benefits of liver transplantation with higher risk of cardiovascular diseases (CVD), chronic kidney diseases (CKD), malignancy and infections.^10^ New onset diabetes mellitus develops in 35.8% of PTMS patients and 61.5% need treatment for hypertension as well with its associated long-term complications.^9^

Despite growing epidemic of MS and increasing need for liver transplantation due to NAFLD related cirrhosis, prevalence and impact of PTMS on morbidity and mortality of post liver transplant patients is variably reported in literature and very few studies have addressed this fast-growing issue. With development of multiple liver transplantation centers across the country, increasing number of patients are undergoing this definitive treatment for liver cirrhosis in Pakistan. However, data regarding PTMS in our patients is scarcely available. We planned a study to determine frequency of post-transplant metabolic syndrome in patients of liver transplantation, its pre-transplant predictors and its long-term complications.

## METHODS

This cross-sectional analytical study was conducted at Department of Gastroenterology Shaikh Zayed Post Graduate Medical Institute Lahore from January 2021 till March 2023. Sample size calculated using online calculator OpenEpi®, was 102 keeping 95% confidence level, 5% margin of error and expected frequency of post-transplant metabolic syndrome to be 25%.^8^ We included patients undergoing liver transplantation at Shaikh Zayed Hospital and surviving for at least one year after transplant, via non-probability purposive sampling. Patients with history of pre-transplant ischemic heart disease, cerebrovascular accident, chronic kidney disease with GFR < 60ml/min and those with active alcohol intake of more than 30g/day for males and 20g/day for females after liver transplantation were excluded.

### Ethical Approval:

It was obtained from the Institutional Review Board (Ref No: F.39/NFRC/admn/IRB/96 dated 10.07.2019).

After informed consent, data was extracted from medical record including age, gender, etiology of underlying liver disease, date of liver transplantation, body weight before and after transplantation, height, post-transplant waist circumference, presence of diabetes mellitus, hypertension or hyperlipidemia before and after liver transplantation, medications being taken including immunosuppressive, hypoglycemic, anti-hypertensive and lipid lowering agents.

Patients were invited for follow up in outpatient department During follow up visit, detailed clinical evaluation was carried out for drug compliance, development of new symptoms or signs suggestive of cardiovascular event, TIA, stroke or chronic kidney disease like chest pain, dyspnea, body swelling, body weakness, numbness etc. Fasting venous sample was collected for fasting blood glucose, fasting lipid profile, renal function tests, liver function tests and complete blood count. Patients with diabetes mellitus, hypertension, dyslipidemia and those who developed cardio or cerebrovascular events or renal dysfunction were treated as per standard therapeutic plan through multi-disciplinary care.

Metabolic syndrome was defined as per National Cholesterol Education Program Adult Treatment Panel III (ATP-III).^1^ Presence of three or more of the following five criteria was required for diagnosis of MS:

- Waist circumference > 90 cm for males and > 80 cm for females (Asian Population).
- Fasting plasma glucose ≥ 100 mg/dl or on treatment for diabetes mellitus.
- Blood pressure ≥ 130/85 mm of Hg or on treatment for hypertension.
- Triglycerides ≥ 150 mg/dl.
- HDL < 40 mg/dl in men and < 50 mg/dl in women.

Major cardiovascular events were defined as transient ischemic attack (TIA), cerebrovascular accident (CVA), acute coronary syndrome (ACS), myocardial infarction. Coronary events were confirmed by coronary angiography while diagnosis of TIA or CVA was based on clinical evaluation and imaging study. De novo diabetes mellitus and hypertension were diagnosed as per standard guidelines.

### Statistical Analysis:

Data was analyzed using SPSS®29 (IBM SPSS Inc). Quantitative variables including age, time since liver transplantation, waist circumference etc were described as mean± standard deviation (SD) or Median while numerical and categorical variables were given as percentage. Patients with and those without PTMS and variables before and after liver transplantation were analyzed using student (t) test for continuous variables and chi square X^2^ for categorical variables with normal distribution while Mann Whitney U test was used for non-parametric variables. Odds ratio (OR) and 95% confidence interval were estimated. Linear regression analysis was done for multivariate analysis taking pre transplant variables as independent while development of PTMS as dependent variable. P-value of ≤ 0.05 was considered significant for data interpretation.

## RESULTS

Total number of post liver transplant (LT) patients included were 111 with mean age of 45.2 (±10.45) years and male to female ratio 6.4/1 (96/15). Liver transplant was done at < 40 years of age in 29(26.1%) patients while 9 (8.1%) had surgery at or above 60 years of age. Major etiology for liver cirrhosis was chronic hepatitis C in 64(57.7%) patients, 15 (13.5%) had hepatitis B, 7 (6.3%) were transplanted for acute liver failure, 3 (2.7%) each for autoimmune hepatitis and Wilson’s disease, 2(1.8%) had Budd Chiari syndrome, 9 (8.1%) had diagnosis of NAFLD while in 8 (7.2%) cirrhosis was labelled as cryptogenic.

Diabetes mellitus was present in 15(13.5%) patients before liver transplantation, 11 (9.9%) had hypertension, 48 (43.2%) were obese and 13 (11.7%) were suffering from metabolic syndrome before LT. Post liver transplant duration at follow-up, was more than two years in 103 (92.8%) patients, more than three years in 67(60.4%), more than five years in 34 (30.6%) patients while 12 (10.9%) patients had their transplant surgery more than seven years back.

During post-transplant evaluation 69 (62.2%) patients were obese as per their waist measurement, DM was present in 64(57.7%) patients, 27(24.3%) were hypertensive, 60 (54.1%) had serum triglycerides ≥ 150 mg/dl and serum HDL was less than desired value in 51(45.9%) patients. Post-transplant metabolic syndrome (PTMS) was diagnosed in 60(54.1%) patients. Follow up laboratory results are compared between patients with and those without PTMS in Table-I. Risk of PTMS increases with time after LT as those with more than two years old LT had 55.3% (57/103) PTMS, with more than three years post LT, PTMS was diagnosed in 56.7% (38/67) and 64.7% (22/34) of patients with more than five years post LT time had PTMS.

**Table-I:** Comparison of patients with and those without Post liver transplant metabolic syndrome (PTMS).

Variables	Patients with PTMS Mean (± SD)	Patients without PTMS Mean (± SD)	Significance (P-value)
Hemoglobin (g/dl)	12.31 (2.08)	12.55 (2.46)	0.21
Platelet (x 10^9^/L)	167.13 (63.7)	169.06 (73.7)	0.29
Prothrombin time (sec)	12.53 (1.83)	13.61 (5.91)	0.33
Creatinine (mg/dl)	1.36 (0.94)	1.20 (0.60)	0.09
Bilirubin (mg/dl)	1.14 (1.28)	1.66 (2.9)	0.02
Albumin (g/dl)	3.42 (0.70)	3.79 (0.72)	0.25
Triglycerides (mg/dl)	202.9 (193.1)	134.3 (86.62)	<0.001*
LDL (mg/dl)	121.1 (48.2)	94.01 (36.50)	0.002*
HDL (mg/dl)	36.8 (9.34)	37.72 (8.64)	0.41
ALT (IU/ml)	80.5 (119.1)	40.2 (26.15)	0.20*
AST (IU/ml)	71.24 (93.76)	38.9 (19.7)	0.18*
ALP (U/ml)	310.01 (350.3)	172.29 (119.1)	0.002*

* Mann Whitney U test, LDL: Low density lipoprotein, HDL: High density lipoprotein, ALT: Alanine aminotransferase, AST: Aspartate aminotransferase, ALP: Alkaline phosphatase.

Post liver transplantation, 40 (33.3%) patients developed anastomotic strictures needing intervention during first six months, significantly more (P-value 0.03) in patients who later developed PTMS 26 (43.3%) as compared to those with no PTMS, 14 (27.4%) during follow up. Ischemic heart disease developed in 7 (6.3%) patients after LT, 30 (27.02%) patients had renal insufficiency with impaired renal function tests and only 7 (6.3%) patients had CVA after liver transplantation. We found no significant difference in frequency of ischemic heart disease (P-value 0.34), chronic kidney disease (P-value 0.73) or CVA (P-value 0.34) between patients with and those without PTMS in our study. Patients who had DM before LT were found to have higher odds for developing PTMS, [OR 15.21 (95% CI 1.92-120.34) P-value < 0.001]. Similarly, those with pre-LT hypertension [OR 10.0(95% CI 1.23-81.06) P-value 0.01] and pre-transplant metabolic syndrome, [OR 12.50 (95%CI 1.56-99.86) P-value 0.003] are more likely to develop PTMS as well. Pre LT obesity was not significantly associated with risk of developing PTMS (P-value 0.11).

PTMS was regressed on pre transplant DM, THN and MS which had significant association on univariate analysis. Model comprising of these independent variables significantly predicts development of PTMS after LT (F (3,107= 3.87, P-value=0.011). However, when coefficient was determined to assess independent influence of each variable, only DM depicted significantly positive impact on development of PTMS (Beta 0.357, t 1.51, p=0.03), Table-II.

**Table-II:** Multivariate Regression analysis for predicting development of Post Liver Transplant Metabolic syndrome.

Regression weight	Beta value	t value	P-value	Result
DM- PTMS	0.352	2.098	0.038	supported
HTN-PTMS	-0.62	-0.367	0.71	Not supported
MS-PTMS	-0.91	-0.982	0.32	Not supported

F (3, 107) = 4.417, DM: Diabetes Mellitus, HTN: Hypertension, MS: Metabolic syndrome, PTMS: Post liver transplant metabolic syndrome.

## DISCUSSION

Nomenclature of NAFLD has recently been modified to emphasize the importance of diagnosis and management of metabolic syndrome in these patients.^11^ NAFLD has been replaced with Metabolic dysfunction associated steatotic liver disease (MASLD), which needs presence of one of cardiometabolic risk factors including Obesity, HTN, DM and deranged lipid profile along with hepatic steatosis, while presence of hepatic inflammation as suggested by cytological ballooning and lobular inflammation along with steatosis and metabolic risk factors will be labelled as metabolic dysfunction associated steatohepatitis (MASH).^12^

We identified alarmingly high prevalence of metabolic syndrome, 54.1% in post liver transplant patients. Ford E et al identified more than two decades back that rate of MS is more than double in post LT patients as compared to age adjusted prevalence of 23.7% in western population.^2^ Prevalence of MS was reported as 59.1% in post-transplant patients as compared to 5.4% before LT by Laish I et al.^13^ Presence of PTMS can result in increased risk for cardiovascular events in post-transplant patients. However, this long-term risk is largely unrecognized as a survey of post liver transplant care-providers identified that only 50% of them felt confident in discussing PTMS and its complications, only one third felt competent in managing it and only 13% reported that their patients had well controlled cardiovascular risk factors of PTMS.^9^

We have noted DM in 57.7% post LT patients as compared to only 13.5% prevalence in same patients before liver transplantation. Hecking M et al concluded that DM is more among LT patients as compared to general population^14^ It is primarily due to post LT medications including steroids, calcineurin inhibitors (CNI), and mTOR inhibitors like sirolimus, which can induce increased glucose output and reduction in insulin production and its sensitivity.^15^ This new onset DM in post-transplant setting is associated with higher long-term mortality especially if not well controlled, moreover its management is also challenging due to continuation of medications responsible for its development.^16^

Hypertension (24.3%), obesity (62.8%) and dyslipidemia (54.1%) were also more prevalent in our post-LT patients as compared to its reported prevalence in general population. Pfitzmann R et al reported hypertension in 49.1% post LT patients who survived for more than five years.^17^ Kuo T et al concluded that around 2/3 patients turn obese after LT due to reversal of cirrhosis, return of appetite and drug induced insulin resistance and have higher long-term mortality.^18^ Dyslipidemia was reported in 46% of post LT patients, immunosuppressive drugs along with dietary habits and sedentary life style are major contributing factors.^19^

We identified presence of DM, Hypertension and Metabolic syndrome before LT as major risk factors for developing PTMS. Hui LW et al noted that old age OR 1.05(95% CI 1.01-1.09, P-value 0.02) and DM before liver transplantation OR 5.0 (95% CI 4.17-5.99, P-value < 0.01) are associated with risk of PTMS.^8^ Perez LV et al concluded that age at LT (OR 1.08; P=0.002), body mass index of before LT (OR 1.23; P < 0.001), serum fasting glucose (OR 1.02; p=0.005) and non-heart beating donor (OR 1.02; p=0.046) are pre transplant variable associated with PTMS.^20^ Presence of MS (OR 2.75, p 0.021), higher fasting plasma glucose (p=0.026) and BMI (p=0.006) were reported as predictor of PTMS by Cai R et al.^21^

Multiple studies have regarded PTMS as risk factor for development of cardiovascular event in post liver transplant patients with reported prevalence as high as 26% after five years.^3^,^7^-^9^ However we failed to identify significant association between cardiovascular events and PTMS in our study. Satapathy SK et al reported that cardiovascular mortality is not different in initial post LT years, however it increases with longer follow up.^22^

Our follow up of patients with PTMS is short as only 30.6% of our study patients had their liver transplanted for more than five years, longer follow up may depict harmful cardiovascular impact of PTMS. Our patients of liver transplantation are from single center which may limit generalizability of our results. Larger data with patients from multiple centers and longer follow up are needed as it may better depict long term implications of developing PTMS.

Presence of PTMS in majority of post liver transplant patients is alarming for healthcare providers, especially those following patients of LT. We are presenting first data on PTMS from our regions where facilities of liver transplantation are in developing phase. It will sensitize our transplant teams that along with due care for post-transplant complications and management of immunosuppressive therapy, attention should be paid to control of cardiometabolic risk factors like obesity, DM, HTN and dyslipidemia through advice for diet control, life style modification, medications or endoscopic or laparoscopic bariatric interventions if needed to avoid long term complications which may mitigate the benefit achieved through successful LT.

## CONCLUSION

Post-transplant metabolic syndrome develops in 54.1% patients after liver transplantation with higher probability for patients with pre liver transplant diabetes mellitus, hypertension and metabolic syndrome. Evaluation for risk of cardiovascular events with PTMS needs longer post liver transplant follow up.

### Author’s contributions:

**ZR:** Conceived and Designed study, data collection, manuscript review and is accountable for accuracy and integrity of data.

**KM:** Conceived the study, Data collection, manuscript review.

**SS:** Designed study, statistical analysis, manuscript writing and is accountable for accuracy and integrity of data.

**AS:** Data collection, analysis, manuscript review.
